# Evaluation of dietary quality in patients with functional gastrointestinal symptoms: a Norwegian single centre study

**DOI:** 10.29219/fnr.v68.10692

**Published:** 2024-12-23

**Authors:** Ida Marie Holm, Sissi Christiane Stove Lorentzen, Monica Hauger Carlsen, Jørgen Valeur, Tonje Mellin-Olsen, Hanna Fjeldheim Dale

**Affiliations:** 1 Department of Clinical Support, Lovisenberg Diaconal Hospital, Oslo, Norway; 2 Department of Nutrition, University of Oslo, Oslo, Norway; 3 Unger-Vetlesen Institute, Lovisenberg Diaconal Hospital, Oslo, Norway; 4 Institute of Clinical Medicine, University of Oslo, Oslo, Norway

**Keywords:** Nutritional evaluation, dietary patterns, irritable bowel syndrome, inflammatory bowel disease, Mediterranean diet, gut-microbiota interaction

## Abstract

**Background:**

Poor dietary quality has been described as a contributor to symptoms in subjects with functional gastrointestinal (GI) symptoms. Hitherto, the focus in dietary evaluation and treatment in this patient group has mainly been on avoiding individual nutrient deficiencies, and less attention has been given to the dietary pattern and the overall food quality. Hence, we aim to describe and evaluate the dietary quality in patients with functional GI symptoms.

**Methods:**

Patients with GI symptoms and a diagnosis of irritable bowel syndrome or inflammatory bowel disease in remission, consecutively referred to a clinical dietitian for nutritional guidance, were included. All participants completed a 7-day weighed food record. The intake of foods, energy, macro- and micronutrients was computed. Dietary quality was evaluated by intake frequencies based on a predefined food index, combined with assessing achievement of nutrient intake recommendations.

**Results:**

A total of 35 patients were included. Intake frequencies of red meat, cheese and sweets were high, whereas intake frequencies of green leafy vegetables, berries, nuts, whole grain and legumes were low. The total intake of vegetables, fruit, berries, fish and nuts was lower than current recommendations, and the intake corresponded to intake below recommendations for several micronutrients, including vitamins D, C and A; iodine; folate; potassium; and selenium.

**Conclusion:**

The group of patients with GI symptoms had overall inadequate dietary quality. Low intake of nutrient-dense food groups considered as beneficial for health corresponded with insufficient intake of several micronutrients. We recommend that dietary evaluation should focus on the intake of food groups, rather than nutrients, in the treatment of patients with functional GI symptoms, to ensure a better evaluation of dietary quality.

## Popular scientific summary

- Dietary evaluation of patients with functional gastrointestinal symptoms has been focusing mainly on the intake of energy and nutrients, whilst less attention has been given the dietary pattern and the overall dietary quality.
- We evaluated the diet of 35 patients with functional gastrointestinal symptoms and found that the patients had overall inadequate dietary quality.
- We recommend that dietary evaluation should focus on the intake of food groups, rather than isolated nutrients, in the current patient group.

Functional gastrointestinal (GI) symptoms are common in patients with irritable bowel syndrome (IBS) and inflammatory bowel disease (IBD) (1, 2). These are gut-microbiota-related disorders, and diet is a central contributor to both risk of disease and generation of symptoms (3–5). Diets with poor dietary quality, such as the ‘Western diet’ with higher intake of fast food and ‘lower quality’ foods such as red meat, sugary and ultra-processed foods have been associated with the severity of symptoms and negative alterations in the gut microbiota in this patient group (6–10). In contrast, dietary patterns in line with the Mediterranean diet have been associated with beneficial gut-microbiota alterations and improvement in GI symptoms (10–12).

Dietary management of functional GI symptoms commonly includes restriction or elimination of specific foods or food groups (13). Elimination diets are associated with risk of nutrient deficiencies and unhealthy dietary patterns, unless supported by appropriate guidance (14, 15). Patients with IBS and IBD are also known to exhibit food-avoidance behaviour and follow self-imposed dietary restrictions (16–18), which highlights the importance of ensuring nutritional adequacy in the treatment of this patient group.

To date, the traditional evaluation of diet has been focusing mainly on the intake of energy, macro- and micronutrients, whilst less attention has been given the dietary pattern and the overall dietary quality in patients with functional GI symptoms (10). The traditional evaluation approach to dietary assessment, which focuses mainly on assessing individual nutrient deficiencies and comparing it with reference values intended for a healthy population, can be regarded as limited. Implementing knowledge about the diet-gut microbiota interaction by considering the overall dietary quality, rather than adjusting individual dietary components, may help optimise dietary treatment in patients with functional GI symptoms (19, 20). Hence, including scores for dietary diversity compared with a reference diet known to be beneficial for this particular patient group may add valuable information when evaluating and adjusting patients’ dietary patterns (21).

Here, we describe and evaluate the dietary quality of patients with functional GI symptoms related to IBS and quiescent IBD, as defined by adherence to a Mediterranean dietary index (MIND diet), combined with achievement of specific nutrient intake recommendations.

## Methods

### Study design

This study was completed between March and December 2021 at the outpatient gastroenterology clinic at Lovisenberg Diaconal Hospital (LDH). Consecutive patients referred to a clinical dietitian with symptoms of IBS according to the Rome IV criteria (22) and a diagnosis of IBS or IBD in remission were eligible for inclusion. Exclusion criteria included: ongoing intestinal inflammation, a diagnosed eating disorder and inability to complete written food records. Participants were recruited during their first appointment with a dietitian at the outpatient clinic, and all participants completed a 7-day consecutive weighed food record (WFR). This study was approved by the data protection officer at LDH, and all participants signed an informed consent form before inclusion.

### Evaluation of dietary quality

The definition of dietary quality varies between studies. In the current study, we evaluate dietary quality using two previously suggested metrics: Dietary diversity (number of different foods consumed) and nutrient adequacy (achievement of recommended intake) (23). Evaluation of dietary diversity was done by calculating intake frequencies according to a predefined food index, based on the MIND diet as defined by Morris et al. (21). The MIND diet is based on the Mediterranean and DASH diets (24) and was selected as the Mediterranean diet is a dietary pattern suggested to be beneficial in the treatment of functional GI symptoms (12). The original definitions from the MIND diet index were revised to include the full variety of foods reported in the WFR. Olive oil and margarine were not captured sufficiently by the WFR and were therefore omitted from the data analysis. We report MIND diet references for optimal frequency intake of food groups, but not the total MIND diet scores. The frequency of intake of the MIND diet food groups was calculated on a weekly basis. Evaluation of nutrient adequacy was calculated based on the dietary intake reported in the WFR and compared with the Norwegian dietary recommendations (25).

### The weighed food record

Participants received a printed WFR booklet together with a digital scale (Beneco, Mod. 9810). They were instructed to weigh all food and drink prior to intake for seven consecutive days. All subjects were as asked to provide detailed descriptions of each food item and weigh and subtract any leftovers. For foods and meals consumed outside of the home, the participants were asked to describe serving sizes using standard household units or other recognisable descriptions.

### Calculation of dietary intake

Total energy intake, food intake and intake of macro- and micronutrients were computed using the food composition database AE-21 and the dietary assessment system *Kostberegningssystemet* (KBS, version 7.4, 2021–2022) developed at the Department of Nutrition, University of Oslo, Norway. Food items or dishes that were missing in the food database were substituted with comparable items/dishes. If no comparable item/dish was found in the database, the nutrition information and/or recipe for the item/dish in question or a comparable item/dish was imported from other food databases, information from food producers, retailers or recipe databases available online. Calculated intake was compared to recommended intake of macro- and micronutrients according to the Norwegian guidelines current in 2022 (25).

### Statistics

Statistical analysis was performed in SPSS (IBM SPSS statistics 28.0.1.1) and Microsoft Excel (Microsoft Excel 16.0). Graphical illustrations were made in GraphPad Prism version 10 (Dotmatics, Boston, USA). Characteristics of the study population are described using descriptive statistics. Categorical variables are presented as frequencies (*n*) and percentages (%). Variables showing normal distribution are presented as mean and 95% confidence interval (95% CI), and variables showing skewed distribution are presented as median and 25th and 75th percentiles (25th, 75th).

## Results

### Baseline characteristics

A total of 58 participants were recruited, of which 11 withdrew and 12 were excluded due to incomplete dietary registration. In total, 35 participants completed the 7-day WFR and were included in the final analysis. The majority of the participants were females diagnosed with IBS. Baseline characteristics are shown in Table 1.

**Table 1:** Descriptive characteristics of participants

Baseline characteristics	All participants, *n* = 35
IBS/IBD	28/7
Female/male	26/9
Age, mean (95% CI)	36.8 (33.2, 40.4)
Body mass index, median (25th, 75th)	24.7 (21.8, 26.3)

IBS, irritable bowel syndrome; IBD, inflammatory bowel disease.

### Evaluation of intake frequencies according to MIND diet index

**Fig. 1:**
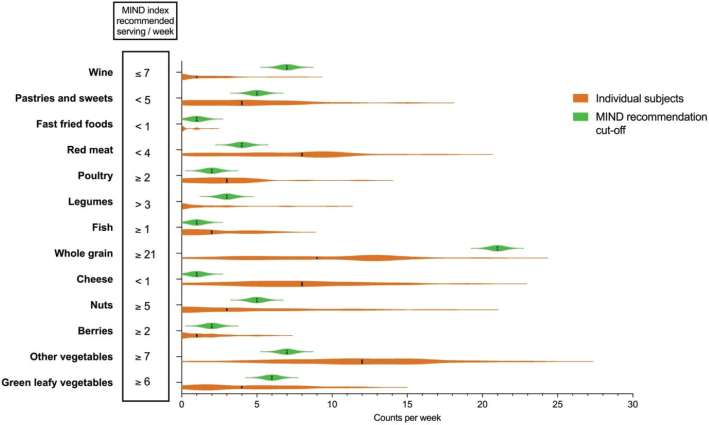
Frequency of intake of different food groups presented as counts per week, as defined in the MIND diet, for the 35 patients with functional gastrointestinal symptoms, including recommended MIND diet cut-offs for each food group (21). The black line on the violin plot shows the median intake.

Frequency counts of intake of different food groups (counted as servings) as defined in the MIND diet and the MIND diet cut-offs values are presented in Fig. 1. The intake of red meat and cheese was reported on average once a day, and the intake of pastries and sweets was reported more than 4 days a week, all higher than what is considered beneficial according to the MIND diet cut-offs. Intake frequency of green leafy vegetables, berries, nuts, whole grain and legumes was lower than what is considered beneficial according to the cut-offs. Intake frequencies of vegetables, fish, poultry, wine and fast fried foods were in accordance with the cut-off values.

### Intake of different food groups

Table 2 describes the total intake of different food groups as calculated from the WFRs. Women had lower median intakes of several of the food groups compared to men. Bread constituted the majority of the participants’ intake of grains, and only a small part of the median intake of vegetables was leafy green vegetables. The intake of both berries and nuts was also low. Participants’ intake of red meat was more than double that of fish, and the median daily intake of legumes was zero. The combined intake of food that can be categorised as ‘pastries and sweets’ (cakes, pastries, biscuits, chocolate and sweets) was 18 g/day.

**Table 2:** Median daily food intake from the weighed food record, in grams per day, amongst all participants, women and men

Food group	Intake*, g/d					
	All participants, *n* = 35		Women, *n* = 26		Men, *n* = 9	
	Median	25th, 75th	Median	25th, 75th	Median	25th, 75th
Bread	83	39, 119	69	31, 113	195	91, 262
Breakfast cereals	6	0, 25	6	0, 24	0	0, 25
Rice	13	0, 52	13	0, 50	20	0, 62
Pasta	0	0, 22	0	0, 22	0	0, 28
Potatoes	31	11, 44	20	10, 42	41	37, 49
All vegetables	147	71, 202	153	104, 222	135	62, 171
**Leafy vegetables**	6	0, 17	1	0, 13	17	6, 25
Fruit	47	15, 133	45	16, 98	129	10, 185
Berries	3	0, 19	4	0, 18	3	0, 28
Juice and smoothies	44	2, 110	47	6, 112	40	1, 129
Nuts	5	0, 25	5	0, 30	6	0, 15
Meat, red	57	26, 88	47	25, 71	57	23, 172
Meat, white	30	18, 48	27	7, 48	31	2, 92
Fish	25	14, 46	23	13, 42	38	32, 83
Eggs	20	0, 47	20	0, 51	9	0. 47
Legumes	0	0, 7	0	0, 7	4	0, 22
Milk and dairy products	251	135, 331	252	113, 494	245	137, 308
**Fermented products**	0	0, 72	13	0, 91	0	0, 54
Cheese	41	21, 59	38	20, 58	42	9, 72
Margarine, oils	5	1, 11	4	1, 9	13	2, 17
Butter	1	0, 5	0	0, 6	4	0, 4
Cakes, pastries and biscuits	6	0, 23	6	0, 24	2	0, 30
Chocolate and sweets	12	4, 33	11	3, 29	32	0, 52
Potato crisps	0	0, 16	0	0, 11	14	0, 46
Sugar-sweetened soft drinks	0	0, 32	0	0, 30	0	0, 47
Artificially sweetened soft drinks	29	0, 165	32	0, 200	0	0, 84
Coffee	186	34, 301	141	5, 230	299	238, 586
Tea	26	0, 260	18	0, 244	26	0, 427
Beer	47	0, 143	0	0, 143	94	47, 329
Wine	29	0, 93	59	0, 130	0	0, 57

* Estimates are based on dietary intake from the weighed food records and presented as median and 25th and 75th percentiles due to skewed distributions of data.

### Intake of energy and macronutrients

The intake of energy and macronutrients is presented in Table 3. The average energy intake was 8.0 MJ per day. The intake of saturated fatty acids was higher than recommended, at 13.2% of total energy intake (E%). Dietary fibre intake was lower than recommended at 22.3 g/day, and the total intake of carbohydrates was also lower than recommended. The average intakes of the remaining macronutrients, including added sugar and alcohol, were in adherence with the Norwegian dietary recommendations.

**Table 3:** Estimated intakes of energy (MJ/day) and energy-providing nutrients (E%) for all study participants, women and men

Nutrient	Estimated dietary intake*			Recommended daily intake**
	All participants, *n* = 35	Women, *n* = 26	Men, *n* = 9	
**Mean (95% CI)**				
Energy, MJ/d	8.0 (7.4, 8.6)	7.4 (6.7, 8.1)	9.7 (8.6, 10.7)	
Protein, E%	17 (16, 18)	17 (16, 19)	17 (15, 18)	10–20
Saturated fat, E%	13 (12, 14)	13 (12, 15)	13 (12, 15)	<10
Carbohydrates, E%	38 (36, 40)	38 (35, 40)	38 (34, 42)	45–60
Dietary fibre, g/d	22 (19, 25)	21 (15, 28)	25 (16, 33)	≥25***
**Median (25th, 75th)**				
Fat, E%	41 (34, 43)	40 (33, 43)	41 (37, 43)	25–40
Monounsaturated fat, E%	16 (13, 17)	15 (12, 17)	17 (14, 20)	10–20
Polyunsaturated fat, E%	6.5 (5, 7.3)	6.6 (5.6, 7.3)	6.4 (5.0, 9.8)	5–10
Omega-3, E%	1.2 (0.8, 1.8)	1.1 (0.8, 1.8)	1.2 (0.9, 1.8)	≥0,5
Added sugar, E%	3.3 (2.3, 5.7)	3.3 (2.3, 4.8)	4.0 (2.7, 7.8)	<10
Alcohol, E%	2.9 (0.6, 7.2)	3.6 (0.9, 8.5)	2.9 (0.3, 6.2)	<5

E%, percentage of total energy intake.

* Intake estimates are based on data from the weighed food records and are presented as mean and 95% CI for normally distributed variables. Variables with skewed distribution are presented as median and 25th and 75th percentiles.

** Intake recommendations for the general adult population from the Norwegian dietary guidelines (25).

*** ≥25 g/day is the minimum recommendation for women, whilst men are recommended ≥35 g/day (25).

### Intake of micronutrients

The tables showing total intakes of micronutrients as well as degree of non-adherence to dietary recommendations for each micronutrient are provided in the Supplementary Material. The intakes of vitamins D, C and A; iodine; folate; potassium; and selenium were lower than recommended for both women and men.

Nearly 75% of participants consumed insufficient amounts of vitamin A. The median intake of vitamin D was 4.8 mg amongst the women and 3.4 mg amongst the men. None of the participants consumed sufficient vitamin D. Whilst the median intake of vitamin C was almost in line with the recommended intake, more than half of the participants reported intakes below recommended levels.

The intake of iodine was 99.3 mg amongst women and 72.9 mg amongst men, with 80% of all participants not meeting recommended intake levels. More than 75% had insufficient intakes of folate, with median intake at 226 mg. More than 50% of participants consumed enough potassium, and over 70% consumed insufficient amounts of selenium. Only 4% of women had sufficient intake of iron, with a median intake of 9.4 mg. Half of the participants had insufficient intakes of calcium. Additionally, half of the participants had higher sodium intakes than recommended.

## Discussion

To our knowledge, this is the first study reporting on dietary quality in patients with GI symptoms by including measures for dietary diversity based on a gut-microbiota-specific beneficial reference diet. The dietary diversity measured by intake frequencies of defined food groups showed that the patients had lower intake frequencies than recommended for several food groups shown to be beneficial for patients with GI disease (12, 21), including green leafy vegetables, berries, nuts, whole grain and legumes. These findings were supported by data on total daily food intake, showing that the intake of vegetables, fruit, berries, whole grain, fish and nuts was low compared with the Norwegian dietary guidelines (25). The reported intake corresponded to intake below recommendations for several micronutrients, including vitamins D, C and A; iodine; folate; potassium; and selenium. Taken together, we found the dietary quality in the group of patients with functional GI symptoms to be inadequate.

For clinical practice, it should be noted that the low reported intakes of micronutrients correspond with low reported intakes of nutrient-dense food groups supplying the micronutrients in question. For example, the low intakes of vegetables, fruit and berries correspond with insufficient intakes of folate and vitamin C. The intake of fish, which was also low, may explain the observed insufficiencies in the intake of vitamin D and iodine. This suggests that a food-based approach can be useful in assessing patients’ diets as well as in the targeting of dietary interventions amongst these patients.

The selection of food groups in this study was based on the wish to investigate the consumptions of foods that may indicate adherence to dietary patterns considered beneficial for patients with functional GI symptoms. As a Mediterranean dietary pattern is suggested to be beneficial in patients with GI symptoms (12, 26), we chose to include the MIND diet index in the current evaluation of dietary quality. Whilst the MIND diet is the only dietary index that uniquely specifies green leafy vegetables and berries, which provides dietary fibre and may be of relevance for the gut microbiota, the clinical relevance of adhering to these food groups has not been investigated (21). Although a study investigating adherence to the MIND diet and the risk of IBS did not find significant associations (27), increasing evidence suggests the use of a Mediterranean dietary pattern in the treatment of already existing GI symptoms (12, 26). The MIND diet classification does not particularly define ultra-processed foods. Ultra-processed foods, including several additives, are suggested to negatively impact the GI tract (10, 28). Notably, it could have strengthened the study to also include the NOVA classification for describing the intake of ultra-processed foods in the current IBS population (29).

Our findings are partly in line with previously reported results on dietary intake in subjects with GI symptoms. A recent study describing dietary intake and nutritional status in newly diagnosed IBD patients reported higher total intake of vegetables, fruit, berries, bread whole grain products, coffee, soft drinks and fish, and lower intakes of milk, cheese and alcohol than what was found amongst the participants in our study (30, 31). Comparable to our results, they reported a high proportion of insufficient dietary intakes of several micronutrients (30). Of note, these data were based on a food frequency questionnaire (FFQ) and not weighed measures; hence, the deviating findings may reflect differences between the dietary assessment methods. Interestingly, a recent cross-sectional observational trial of American adults highlights the importance of a food-based approach when evaluating diet related to GI health (32). They present that higher dietary quality, seen as habitual intake of legumes, vegetables and soluble fibres, is negatively correlated with markers for GI inflammation (32).

The intake of red meat in our population was within the recommendations and low compared to both the general population and IBD patients (25, 30, 33). The low meat intake could correspond with the low iron levels detected; hence, it has to be highlighted that although meat is considered to reduce the food quality in the current evaluation, meat contributes with a lot of beneficial nutrient when eaten in recommended amounts. The intake of sweets was lower than expected compared to the general population (33). The reported intake of dietary fibre was also lower than recommended, corresponding to the low reported intake of bread and other whole grain cereals, vegetables, fruits and berries. Low fibre intake has been reported in other studies on comparable populations and may be related to perceived intolerance to carbohydrate-containing foods, of which intake was also low, and likely related to the reduction of FODMAPs (16, 18, 30, 34). The low fibre intake raises clinical concerns related to both overall health and concerns related to the gut microbiota (35, 36). The dietary intake of saturated fat was higher than recommended but comparable to that of the general population and of other patients with functional GI symptoms (25, 30, 33).

Notably, the average energy intake amongst the participants was generally low compared to estimated energy needs. Keeping food records and weighing food items are known to cause dietary change due to self-monitoring, particularly with multiple recording days, and underreporting is particularly common for foods considered as unhealthy (37, 38). It is likely that underreporting may have affected our results, and that the participants in fact have diets of even worse quality than captured by the WFR, as unhealthy foods are likely underreported. In addition, food-avoidance is a known issue in patients with functional GI symptoms and may be particularly likely in a sample of untreated patients (16, 18), which might explain the low intakes reported for several food groups, such as legumes, nuts and vegetables. The intake frequency of MIND diet food groups that are considered beneficial was calculated even from ultra-processed foods, for example, tomato sauce counting as ‘vegetables’ although present in an ultra-processed dish such as ready-bought pizza, lasagna or pasta. Ultra-processed foods constitute a large share of peoples diets today, and there is concern regarding the role of ultra-processed foods in affecting dietary quality, as well as in the etiology/symptom generation of functional GI symptoms, which have yet to be explored sufficiently (26, 39).

In conclusion, our findings show that the group of patients with GI symptoms investigated had overall inadequate dietary quality. Low intake of nutrient-dense food groups considered as beneficial for health, including vegetables, fruit, berries, whole grain, nuts, legumes and fish corresponded with insufficient intake of several micronutrients. We recommend that dietary evaluation should focus on the intake of food groups, rather than nutrients, in the treatment of patients with functional GI symptoms, to capture a better evaluation of dietary quality.

## Supplementary Material


