# Supplementary material for: Evaluation of dietary quality in patients with functional gastrointestinal symptoms: a Norwegian single centre study
Source: Food Nutr Res. 2024 Dec 23;68:10.29219/fnr.v68.10692. doi: 10.29219/fnr.v68.10692 (PMC11708516; doi:10.29219/fnr.v68.10692)
Supplement: Supplementary file 1 [file FNR-68-10692-s1.pdf]

Supplementary Material

Table 1. Estimated daily intakes of vitamins, minerals and trace elements for all study participants, women and men.

**Micro-nutrient**	**Intake***						**Recommended intake****
	**All participants, n = 35**		**Women, n = 26**		**Men, n = 9*****		Women/Men
	Median	*25th, 75th*	*Median*	*25th, 75th*	*Median*	*25th, 75th*	
**Vitamin A, mcg**	591	369, 754	562	368, 700	654	366, 1060	700/900
**Retinol, mcg**	391	268, 548	359	249, 496	522	322, 871	
**Beta-carotene, mcg**	1834	1093, 2663	1912	1220, 3073	1206	530, 2115	
**Vitamin D, mcg**	4.8	3.1, 6.0	4.8	3.2, 6.0	3.4	2.2, 7.2	10
**Vitamin E, mg**	13	10, 17	13	10, 15	15	11, 24	8/10
**Thiamine, mg**	1.3	1.0, 1.6	1.2	0.9, 1.4	1.7	1.3, 1.9	1.1/1.4
**Riboflavin, mg**	1.5	1.1, 1.8	1.4	1.0, 1.8	1.7	1.3, 1.8	1.3/1.7
**Niacin, mg**	167	13, 22	15	12, 21	20	18, 29	15/18
**Vitamin B6, mg**	1.6	1.3, 2.0	1.5	1.2, 1.9	1.8	1.5, 2.1	1.2/1.5
**Folate, mcg**	226	185, 288	215	183, 290	245	207, 313	300
**Vitamin B12, mcg**	5.3	3.9, 6.1	5.2	4.0, 6.3	5.5	3.8, 6.2	2
**Vitamin C, mg**	71	55, 106	73	60, 115	71	48, 96	75
**Calcium, mg**	793	541, 1013	806	533, 1024	793	629, 945	800
**Iron, mg**	9.7	7.4, 11.3	9.4	6.9, 10.6	12.3	8.7, 13.0	15/9
**Sodium, g**	2.6	2.2, 2.9	2.5	2.1, 2.8	2.8	2.4, 4.0	2.4
**Potassium, g**	3.0	2.5, 3.5	3.0	2.1, 3.5	3.3	2.9, 3.5	3.1/3.5
**Magnesium, mg**	323	252, 434	314	228, 405	327	323, 477	280/350
**Zinc, mg**	9.4	7.5, 11.1	8.7	6.6, 10.1	11.1	9.4, 14.0	7/9
**Selenium, mcg**	44.0	35.0, 58.0	41.0	35.0, 53.8	51.0	39.5, 61.5	50/60
**Copper, mg**	1.3	0.9, 1.7	1.2	0.8, 1.5	1.3	1.2, 1.9	0.9
**Phosphorous, mg**	1424	1154, 1828	1294	1028, 1755	1598	1397, 1942	600
**Iodine, mg**	94	64, 145	99	65, 156	73	60, 106	150

* Estimates are based on dietary intake, excluding supplements, from the weighed food records and presented as median and 25th and 75th percentiles.
**Intake recommendations for the general adult population from NNR 2012 (23).
***The sample contains a low number of men, i.e. there is a large degree of uncertainty associated with the estimates.

Table 2. Percentage (%) of all participants, women and men whose dietary intake of vitamins, minerals and trace elements did not meet the recommended daily intake levels.

Micronutrient	Non-compliance with dietary recommendations*, %		
	All participants, n = 35	Women, n = 26	Men, n = 9**
**Vitamin A**	74	77	67
**Vitamin D**	100	100	100
**Vitamin E**	11	12	11
**Thiamine**	34	39	22
**Riboflavin**	49	46	56
**Niacin**	43	50	22
**Vitamin B6**	26	23	33
**Folate**	77	77	78
**Vitamin B12**	3	4	0
**Vitamin C**	57	54	67
**Calcium**	51	50	56
**Iron**	77	96	22
**Sodium**	63	58	78
**Potassium**	57	58	56
**Magnesium**	43	39	56
**Zinc**	29	31	22
**Selenium,**	71	69	78
**Copper**	29	35	11
**Phosphorous**	0	0	0
**Iodine**	80	77	89

*Intake recommendations for the general adult population from NNR 2012 (23).
**The sample contains a low number of men, i.e. there is a large degree of uncertainty associated with the estimates.
